# Fracture strength of CAD/CAM glass fiber post-and-cores: a systematic review

**DOI:** 10.2340/biid.v13.46724

**Published:** 2026-08-19

**Authors:** Amira Yousfi, Rihab Dakhli

**Affiliations:** Department of Prosthodontics, Faculty of Dental Medicine, University of Monastir, Monastir, Tunisia

**Keywords:** CAD/CAM, glass fiber post-and-core, fracture strength, endodontically treated teeth, fiber-reinforced composite, post-and-core

## Abstract

**Background/Objective(s)/Introduction:**

Endodontically treated teeth with extensive coronal tissue loss often require post-and-core restorations to provide retention and support for definitive restorations. Recent advances in computer-aided design/computer-aided manufacturing (CAD/CAM) technology have enabled the fabrication of customized glass fiber post-and-core systems with improved adaptation to root canal anatomy. However, evidence regarding their fracture strength remains inconsistent. This systematic review aimed to evaluate the fracture strength of CAD/CAM-fabricated glass fiber post-and-core systems, compare their performance with that of other post systems, and identify the factors influencing their fracture strength.

**Materials and Methods:**

A systematic review was conducted according to PRISMA guidelines and registered in PROSPERO (CRD420251038278). Electronic searches were performed in PubMed, Scopus, Web of Science, and Cochrane Library in January 2025 and updated in February 2026. Studies evaluating the fracture strength of CAD/CAM glass fiber post-and-core systems were included. Data regarding study characteristics, materials, testing methods, aging procedures, fracture strength outcomes, failure modes, and principal findings were extracted. Methodological quality was assessed using a modified CONSORT checklist adapted for in vitro studies.

**Results:**

A total of 17 studies met the eligibility criteria. Fracture strength values varied considerably according to material composition, testing protocols, and experimental conditions. CAD/CAM glass fiber post-and core systems generally demonstrated fracture strength comparable to cast metal and other contemporary post systems. Improved adaptation to root canal anatomy and thinner cement layers were consistently reported. Although some materials exhibited higher fracture strength values, CAD/CAM glass fiber posts were frequently associated with more favorable and repairable failure modes.

**Conclusion(s):**

CAD/CAM-fabricated glass fiber post-and-core systems represent a promising alternative to conventional post systems, providing fracture strength comparable to established restorative options while promoting more favorable failure behavior. However, methodological heterogeneity among studies highlights the need for standardized testing protocols and further clinical investigations.

**KEY MESSAGES**

1. CAD/CAM-fabricated glass fiber post-and-core systems demonstrate fracture strength comparable to conventional post systems in endodontically treated teeth.
2. Improved adaptation to root canal anatomy and reduced cement thickness are among the principal advantages of CAD/CAM-fabricated glass fiber post-and-core restorations.
3. CAD/CAM glass fiber post-and-core systems are frequently associated with more favorable and repairable failure modes than rigid post materials.

## Introduction

Endodontically treated teeth are particularly vulnerable to biomechanical failure because of the extensive destruction of dental tissues caused by caries, endodontic procedures, and restorative interventions. The resulting decrease in coronal and radicular dentin compromises the structural integrity of the tooth and increases its susceptibility to fracture under functional loading. When the remaining coronal structure is insufficient to retain the definitive restoration, intraradicular posts are commonly indicated to improve retention and contribute to stress distribution within the tooth-restoration complex [1].

Cast metal post-and-core systems have long been considered a reliable restorative option because of their favorable retention and intimate adaptation to root canal anatomy [2]. Nevertheless, their high elastic modulus promotes stress concentration within the root dentin, increasing the risk of catastrophic root fractures [3]. In addition, their metallic color may compromise esthetic outcomes, particularly in the anterior region [4].

Fiber-reinforced posts were introduced to overcome some of these limitations. Owing to their elastic modulus being closer to that of dentin, these posts provide a more favorable distribution of functional stresses and reduce stress concentration within the root structure [3, 5, 6]. Despite these advantages, prefabricated fiber posts often demonstrate limited adaptation in enlarged, flared, or irregular root canals. Consequently, thick cement layers or relining procedures are frequently required, which may adversely affect the integrity of the adhesive interface. Furthermore, relining procedures create multiple interfaces between the post and the composite resin, while the highly cross-linked surface of prefabricated posts may compromise chemical bonding with the relining material [7].

The continuous evolution of computer-aided design and computer-aided manufacturing (CAD/CAM) technology has introduced new possibilities for fabricating customized post-and-core systems through digital workflows, utilizing direct intraoral optical scanning or indirect digitization to achieve high-precision post space acquisition [8, 9].

Compared with prefabricated fiber posts, CAD/CAM technology enables the fabrication of anatomically adapted, one-piece restorations from industrially polymerized fiber-reinforced blocks [7]. Depending on the fabrication material and manufacturing technique, these customized restorations may improve canal wall adaptation and reduce cement thickness, thereby optimizing frictional retention, which may in turn enhance the overall biomechanical performance of the restored tooth [10–12].

In addition, CAD/CAM glass fiber-reinforced materials combine favorable esthetics with mechanical properties closer to those of dentin because of their fiber architecture and relatively low elastic modulus [5, 6]. Although these developments have expanded the clinical applications of CAD/CAM post-and-core systems, questions remain regarding their mechanical performance under functional loading. A recent systematic review examined the influence of fabrication material and manufacturing techniques on the internal adaptation of customized post-and-core restorations [11]. However, evidence specifically addressing the fracture strength of CAD/CAM-fabricated glass fiber post-and-core systems remains limited, and published studies report inconsistent findings [13–15]. Therefore, a comprehensive synthesis of the current literature is warranted to clarify the available evidence regarding their fracture strength and failure behavior.

Accordingly, this systematic review aimed to evaluate the fracture strength of CAD/CAM-fabricated glass fiber post-and-core systems, compare their performance with that of other post systems, and identify the factors influencing their fracture strength.

## Materials and methods

### Protocol registration and reporting guidelines

This systematic review was conducted in accordance with the Preferred Reporting Items for Systematic Reviews and Meta-Analyses (PRISMA) 2020 guidelines [16]. The review protocol was prospectively registered in the International Prospective Register of Systematic Reviews (PROSPERO; registration number: CRD420251038278).

The original electronic search was completed in January 2025 as part of the initial version of the review. To update the available evidence prior to publication, an additional search was conducted in February 2026 to identify newly published eligible studies. No modifications were made to the original protocol, eligibility criteria, outcomes, or review objectives during the update process.

### Research question

The research question of this systematic review was developed according to the PICO framework:

- **Population (P):** Endodontically treated teeth restored with post-and-core systems
- **Intervention (I):** CAD/CAM-fabricated glass fiber post-and-core
- **Comparison (C):** Conventional prefabricated fiber posts and other post systems
- **Outcome (O):** Fracture strength

### Eligibility criteria

#### Inclusion criteria

Studies were included when they fulfilled the following criteria:

1. Investigated CAD/CAM fabricated glass fiber post-and-core systems.
2. Evaluated fracture strength and/or related mechanical properties.
3. Were original in vitro experimental studies.
4. Were published in English and available in full text.
5. Were published between January 2015 and February 2026.

### Exclusion criteria

The following studies were excluded:

1. Case reports, case series, narrative reviews, systematic reviews, and meta-analyses.
2. Conference abstracts, editorials, and letters to the editor.
3. Clinical studies, retrospective observational studies, finite element analyses, and animal studies.
4. Studies that did not report fracture strength outcomes or provided insufficient methodological information.
5. Articles published in languages other than English.

### Search strategy

A systematic electronic search was conducted in PubMed, Scopus, Web of Science, and the Cochrane Library to identify relevant studies evaluating the fracture strength of CAD/CAM glass fiber post-and-core. The initial search was performed in January 2025 and was subsequently updated in February 2026 to identify recently published eligible studies prior to manuscript submission. A gray literature search was also conducted; however, no additional eligible studies were identified. In addition, the reference lists of the included studies were manually screened to identify potentially eligible articles that may not have been retrieved through the electronic search.

The search strategy combined Medical Subject Headings (MeSH) and free-text terms related to CAD/CAM technology, glass fiber posts, post-and-core restorations, and fracture strength. Boolean operators (‘AND’ and ‘OR’) were used to optimize the search process. The main search terms included ‘CAD/CAM’, ‘Computer-Aided Design’, ‘glass fiber post’, ‘glass fiber post and core’, ‘post and core’, ‘fracture resistance’, ‘fracture strength’, and ‘flexural strength’. The search syntax was adapted according to the requirements of each database. The detailed search strategies for all databases are presented in Appendix.

### Study selection

**Figure 1:**
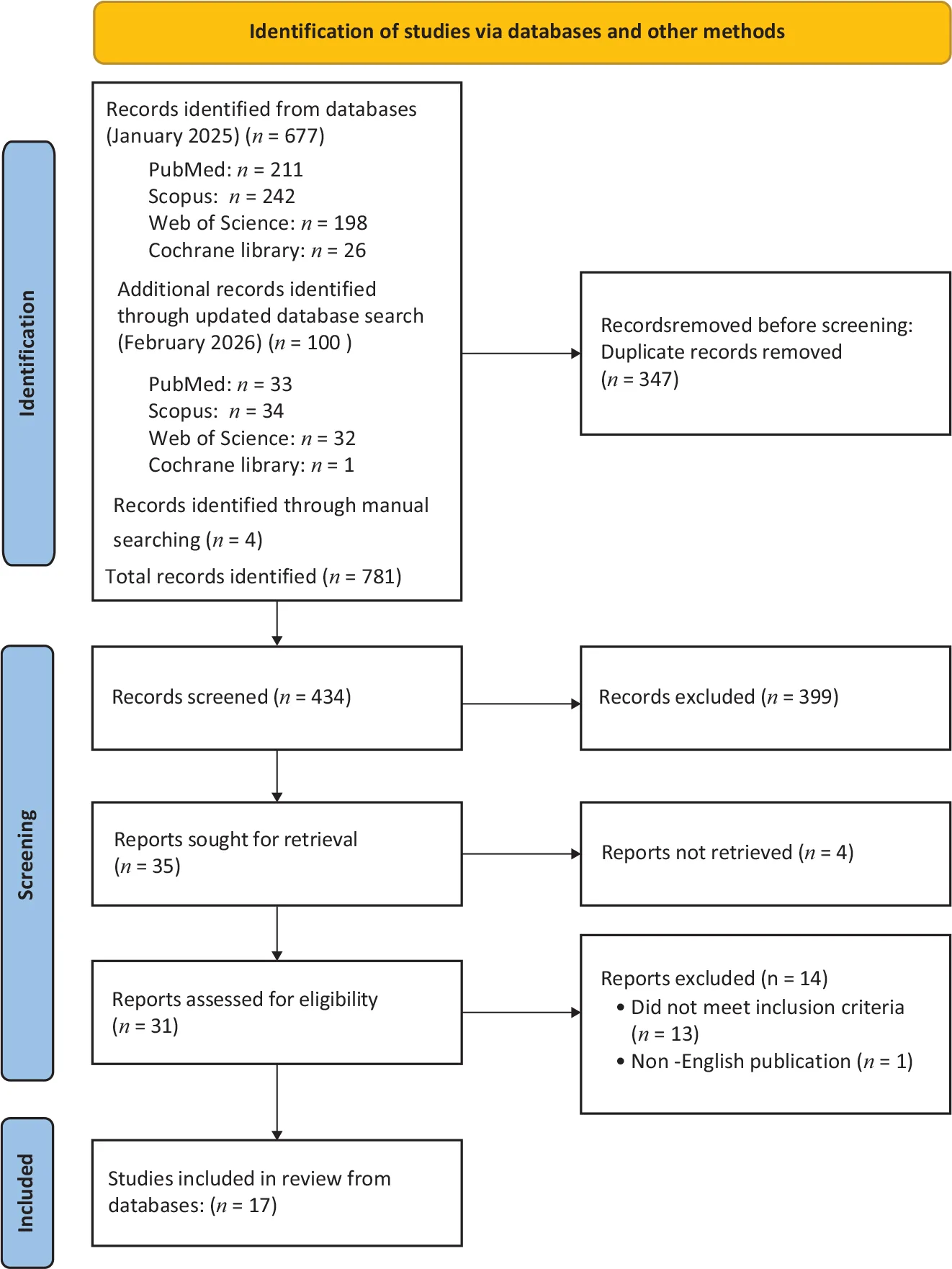
PRISMA 2020 flow diagram of the literature search and study selection process.

All retrieved records were imported into Rayyan for duplicate removal and screening management. Duplicate studies were initially removed automatically using the software algorithm and subsequently verified manually to ensure accuracy. Two independent reviewers (A.Y. and R.D.) screened the titles and abstracts according to the predefined eligibility criteria. Studies considered potentially relevant underwent full-text assessment to determine final eligibility. Any disagreements were resolved through discussion until consensus was reached. The complete study selection process, including the updated search conducted in February 2026, was documented using a PRISMA 2020 flow diagram (Figure 1).

### Data extraction

Two independent reviewers (A.Y. and R.D.) performed data extraction using standardized extraction forms. The extracted data included the author and year of publication, tooth type, sample size, materials and comparison groups, aging procedures, testing methods, fracture strength outcomes, failure modes, and the principal findings of each study. Any discrepancies were resolved through discussion until consensus was reached.

### Risk of bias assessment

For the included in vitro studies, methodological quality was assessed using a modified CONSORT checklist for in vitro studies proposed by Faggion [17]. Although the CONSORT statement was originally developed as a reporting guideline rather than a dedicated risk-of-bias tool, the modified checklist has been adopted in several systematic reviews of laboratory studies to assess methodological quality. Therefore, this checklist was considered an appropriate and standardized method for evaluating the methodological quality of the included in vitro studies.

**Figure 2:**
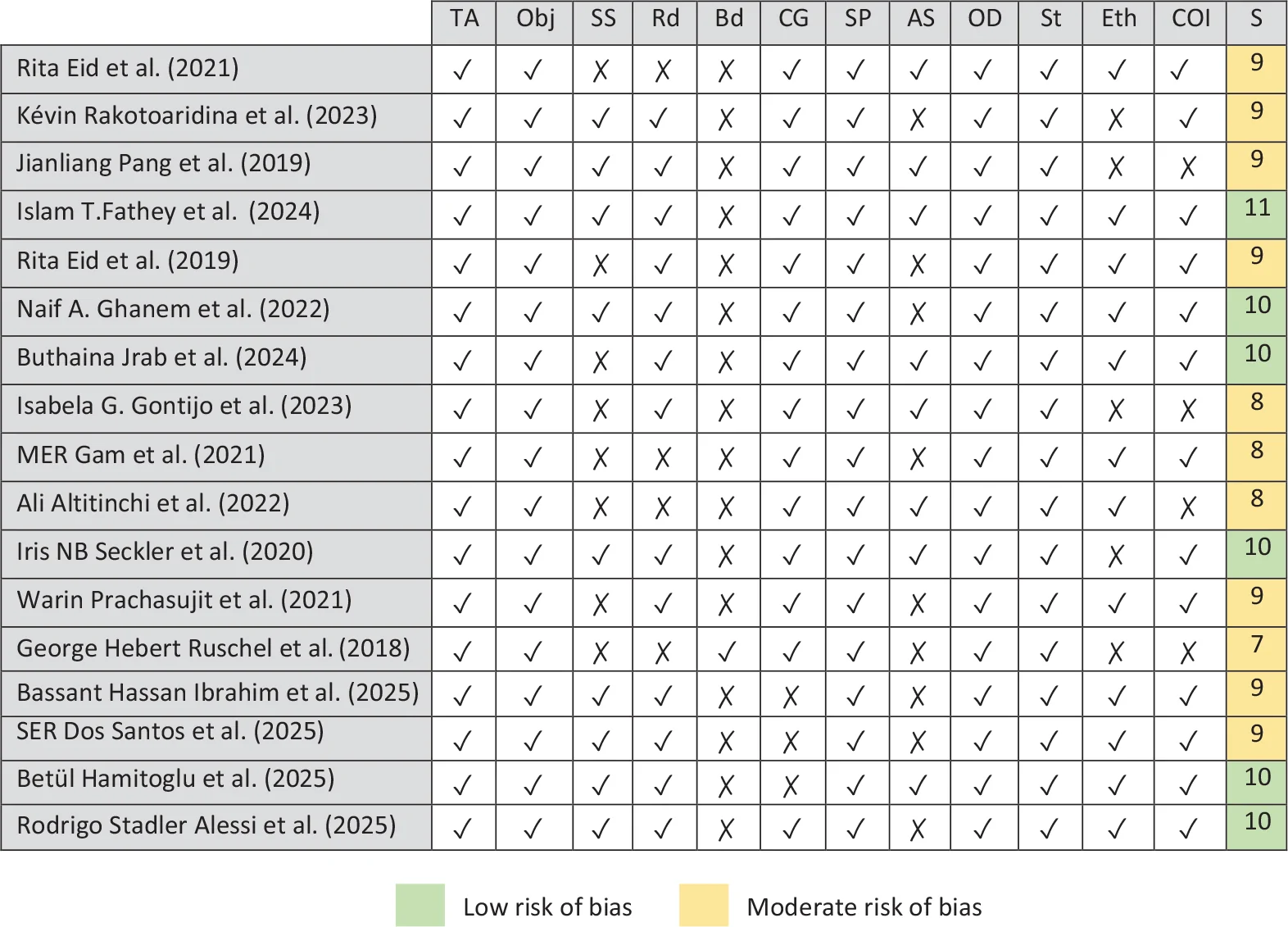
Risk of bias assessment of the included studies based on the modified CONSORT criteria. TA: title and abstract clarity; Obj: objective clearly stated; SS: sample size justification; Rd: random allocation; Bd: blinding described; CG: control group present; SP: standardized procedure; AS: aging simulation used; OD: outcome clearly defined; St: statistical analysis reported; Eth: ethics statement reported; COI: conflict of interest declared; S: total score.

Each study was evaluated across 12 methodological items and scored as ‘Yes’ or ‘No’ according to whether the criterion was fulfilled. Based on the total score, studies were categorized as presenting low [10–12], moderate [7–9], or high (≤ 6) risk of bias. The detailed risk of bias assessment of the included studies is presented in Figure 2.

### Data synthesis

Because of the methodological heterogeneity among the included studies regarding tooth type, CAD/CAM materials, comparator post systems, ferrule design, cementation protocols, aging procedures, fracture testing protocols (including loading angle and crosshead speed), and reported outcomes, quantitative meta-analysis was not considered appropriate. Moreover, outcome measures were reported using different testing conditions and methodologies, further limiting direct quantitative comparison across studies. Subgroup analyses were also deemed infeasible because the number of studies within comparable subgroups was insufficient to provide reliable pooled estimates.

Therefore, a qualitative narrative synthesis was performed. The findings were analyzed descriptively, with particular emphasis on the factors influencing fracture strength and failure behavior of CAD/CAM fiber post-and-core systems and their potential clinical implications.

## Results

### Study selection

The literature search conducted in January 2025 identified 677 records from four electronic databases, including PubMed (*n* = 211), Scopus (*n* = 242), Web of Science (*n* = 198), and the Cochrane Library (*n* = 26). An updated search performed in February 2026 yielded an additional 100 records, comprising 33 from PubMed, 34 from Scopus, 32 from Web of Science, and one from the Cochrane Library. Four additional records were identified through manual searching, resulting in a total of 781 records.

After the removal of 347 duplicate records, 434 studies remained for title and abstract screening. Of these, 399 records were excluded for not meeting the eligibility criteria. Thirty-five reports were sought for full-text retrieval, of which four could not be obtained. The remaining 31 full-text articles were assessed for eligibility. Fourteen studies were excluded following full-text evaluation, including 13 studies that did not meet the inclusion criteria and one study published in a language other than English. Ultimately, 17 studies were included in the qualitative synthesis. The study selection process is illustrated in the PRISMA 2020 flow diagram (Figure 1).

### Characteristics of included studies

The 17 included studies were published between 2018 and 2025 and consisted exclusively of in vitro studies [5–7, 10, 13–15, 18–27]. Sample sizes ranged from 5 to 15 specimens per experimental group. Various CAD/CAM-fabricated post-and-core systems were evaluated, including glass fiber-reinforced composite, polyetheretherketone (PEEK), polyetherketoneketone (PEKK), polymer-infiltrated ceramic network (PICN), zirconia, and high-density polymer materials. Most studies compared CAD/CAM-fabricated posts with conventional prefabricated fiber posts, cast metal post-and-core systems, or other CAD/CAM restorative materials.

Fracture strength was commonly assessed using universal testing machines under static compressive loading. Loading angles ranged from 45° to 150°, while crosshead speeds varied between 0.5 and 2 mm/min. Several studies incorporated artificial aging procedures, including thermocycling, cyclic mechanical loading, or combined thermomechanical aging, to simulate clinical conditions. Detailed characteristics of the included studies are presented in Table 1.

**Table 1:** Data extraction for in vitro studies.

S. No	Author (year)	Groups/Materials evaluated	Sample Size (n)/Teeth type	Testing method	Aging Procedure	Fracture Strength	Failure Mode	Main findings
1	Rita Eid et al. (2021) [10]	1. CPC 2. MFP (Trilor) 3. HDP (Ambarino) 4. PICN (Vita Enamic)	10 per group (total 40) – human maxillary central incisors	Universal testing machine, 135° oblique load, 1 mm/min	Thermomechanical: 250,000 cycles (50 N, 2.6 Hz) + 3000 thermal cycles (5-55°C)	~600 N ~580 N ~570 N ~560 N	CAST: 80% crown fracture (type 1). CAD/CAM groups: mostly crown/post fractures (types 1-2). PICN: 20% catastrophic.	No significant difference in fracture resistance among CAD/CAM posts and cast metal. CAD/CAM materials are acceptable for anterior restorations.
2	Kévin Rakotoaridina et al. (2023) [6]	1. LuxaCam PEEK 2. PICN (Vita Enamic) 3. MFP: Numerys GF 4. LuxaPost :PFP	10 per group (total 40) – human maxillary central incisors	Oblique compressive test, 135° angle, 2 mm/min	None	9.48 ± 6.65 MPa 6.05 ± 4.14 MPa 15.35 ± 6.65 MPa 15.88 ± 4.37 MPa	Material fracture rate (post-only + mixed): PEEK: 20% Enamic: 100% Numerys GF: 30% Luxapost: 20%	MFP showed higher fracture resistance than PEEK and Enamic. Enamic was most protective of the root (no tooth fracture).
3	Jianliang Pang et al. (2019) [5]	1. MFP 2. PFP + composite core 3. Cast gold alloy post-core	10 per group (total 30) – human maxillary central incisors	Static loading after fatigue, 45° angle, 1 mm/min	Mechanical: 300,000 cycles (100 N, 6 Hz)	927.6 ± 275.6 N 616.5 ± 154.9 N 967.9 ± 157.5 N	6 repairable, 4 irreparable 7 irreparable 9 irreparable	CAD/CAM glass fiber posts had comparable strength to cast metal, higher than prefabricated fiber posts, and more repairable fractures.
4	Islam T.Fathey et al. (2024) [18]	1. MFP (Trilor) 2. PEEK 3. PICN (Vita Enamic)	13 per group (total 39) – human maxillary central incisors	Universal testing machine, 135° angle, 1 mm/min	Thermocycling (5000x, 5-55°C) + cyclic loading (50,000x, 50 N, 2 Hz)	452.60 ± 105.90 N 286.16 ± 67.09 N 426.76 ± 77.99 N	Restorable (%): 38.5% 46.2% 53.8% (no significant difference)	MFP and PICN showed higher fracture resistance than PEEK. All exceeded normal occlusal forces.
5	Rita Eid et al. (2019) [7]	1. PFP+ composite core 2. MFP: (Trilor) 3. High-density polymer: Ambarino	10 per group (total 30) – human mandibular second premolars	Universal testing machine, vertical load on buccal cusp, 1 mm/min	None	426.08 ± 128.26 N 367.06 ± 72.34 N 620.02 ± 54.29 N	Mixed (core debonding/fracture). Cohesive core crack. Cohesive core fracture (2 with tooth fracture above CEJ)	The high-density polymer exhibited the best fracture resistance. Milling may compromise MFP properties.
6	Naif A. Ghanem et al. (2022) [19]	1. PEKK 2. MFP (Trilor) 3. PFP	10 per group (total 30) – human mandibular premolars	45° shear force on inner slope of buccal cusp, 1 mm/min (with metal crowns)	None	423.2 ± 113.28 N 356.9 ± 141.05 N 439.5 ± 100.62 N	Reparable: PEKK 60%, MFP 80%, PFP 50% PEKK: detachment; MFP: cervical fracture.	No significant differences. Custom-milled PEKK and FRC are acceptable alternatives to prefabricated fiber posts.
7	Buthaina Jrab et al. (2024) [20]	1. Cast metal (control) 2. Vita Enamic (PICN) 3. Shofu HC 4. MFP: Trilor 5. PEKK	15 per group (total 75) – human mandibular second premolars	Compressive force, 1 mm/min	Thermal cycling (5000x, 5-55°C)	244.41 ± 75.2 N 89.67 ± 24.08 N 97.06 ± 28.11 N 109.2 ± 33.06 N 102.56 ± 25.2 N	Unfavorable fractures: metal 100%, others 0-13%	Metal had the highest fracture resistance but all catastrophic failures. CAD/CAM materials had lower strength but favorable failure modes.
8	Isabela G. Gontijo et al. (2023) [21]	1.PFP 2. Anatomic glass fiber post 3. MFP 4. PEEK	10 per group (total 80, with/without aging) – bovine incisor roots	Compression, 45° angle, 0.5 mm/min	Mechanical aging (300,000 cycles, 50 N, 1.2 Hz)	Without aging: 535.8 ± 182.2 N 677.6 ± 236.1 N 723.4 ± 307.9 N 735.2 ± 209.8 N With aging, all are significantly lower	Repairable fractures are more prevalent in MFP and PEEK groups (50-60% without aging, 30-40% with aging)	Milled glass fiber and PEEK post-cores showed higher fracture resistance and more repairable fractures than prefabricated posts.
9	MER Gam et al. (2021) [13]	1. PFP 2. REL (relined with composite) 3. MFP	10 per group (total 30) – human canines (upper/lower)	Load-to-fracture, 45° angle, 1 mm/min	None	610.58 ± 99.21 N 738.13 ± 151.19 N 544.83 ± 135.80 N	Reparable core fractures: MFP 70%. Irreparable: MFP 30%, REL 10%	CAD/CAM MFP had lower load-to-fracture than relined posts but better adaptation (fewer voids) and more repairable fractures.
10	Ali Altitinchi et al. (2022) [22]	1. CPC 2. Zirconia (3Y-TZP) 3. MFP (Trinia)4. PFP	10 per group (total 40)– human maxillary premolars	Mastication simulator, cyclic loading	1.2 million cycles + 10,000 thermal cycles (5-55°C)	No failures No failures Fractures ~ 504,000 cycles Fracture ~ 752,000 cycles	Not reported	Cast metal and zirconia had superior fatigue resistance. MFP fractured earlier than fiber posts.
11	Iris NB Seckler et al. (2020) [23]	1. CPC 2. GFP 3. MFP	10 per group (total 30) – human mandibular premolars	Compression test, 45° angle, 0.5 mm/min	Thermomechanical: 10,000 thermal cycles (5-55°C) + 300,000 mechanical cycles (30 N)	213.58 ± 93.58 N 78.93 ± 31.33 N 184.80 ± 84.79 N	Cohesive dentin failure (unfavorable): CPC 80%. Cohesive core failure: GFP 100%. Adhesive & cohesive core: CAD/CAM 50% each.	CAD/CAM glass fiber posts had comparable fracture resistance to cast metal and were not associated with irreparable root fractures.
12	Warin Prachasujit et al. (2021) [14]	1. Unidirectional experimental disk 2. Multidirectional experimental disk 3. PFP: RelyX (prefabricated) 4. FRC PostecPlus	10 per group (total 40) – no teeth (material testing)	Three-point bending (ISO 14125), 8 mm span, 0.5 mm/min	None	739.1 ± 24.1 MPa 385.4 ± 41.1 MPa 971.8 ± 42.2 MPa 987.3 ± 44.8 MPa	Not applicable (material testing only)	Fiber direction significantly affects mechanical properties. Unidirectional orientation provides higher flexural strength.
13	George Hebert Ruschel et al. (2018) [24]	1. PFP (Exacto) 2. C-Cd (CAD/CAM milled, diagonal) 3. C-Cv (CAD/CAM milled, vertical)	10 per group (total 30) – no teeth (material testing)	Three-point bending, 8 mm span, 0.5 mm/min	None	900.1 ± 30.4 MPa 357.2 ± 30.7 MPa 101.8 ± 4.3 MPa	PFP: green-stick (partial bend fracture). C-Cd: diagonal/delamination. C-Cv: transverse (brittle cross-fracture).	PFP had superior flexural strength. Milling orientation critically affects the mechanical properties of the CAD/CAM glass fiber post.
14	Bassant Hassan Ibrahim et al. (2025) [25]	1. PICN : Vita Enamic 2. PEEK 3. MFP: Trilor Subgroups: 6 mm vs 9 mm post length	7 per subgroup (total 42) – human maxillary central incisors	Universal testing machine, 45° angle, 1 mm/min	None	6 mm: 381.96 ± 10.18 N 470.21 ± 12.83 N 637.02 ± 11.01 N 9 mm: 400.52 ± 11.38 N 509.76 ± 17.19 N 673.63 ± 12.85 N	Unfavorable fractures: Trilor (28.5%), PEEK (21.4%), Enamic (14.2%). The 9 mm showed more unfavorable results than the 6 mm.	Trilor had the highest fracture resistance but the most unfavorable fractures. 9 mm posts had higher strength than 6 mm posts.
15	SER Dos Santos et al. (2025) [15]	1. PFP 2. Anatomic fiber post 3. MFP	5 per group (photoelastic + flexural) – simulated roots (photoelastic resin from human canine)	Three-point bending (flexural) + photoelastic stress analysis	None	123.0 ± 26.9 MPa 230.3 ± 18.9 MPa 50.8 ± 7.9 MPa	Photoelastic stress: lowest in MFP and PFP; highest in the anatomic post.	MFP had lowest flexural strength but favorable stress distribution (lower apical stress).
16	Betül Hamitoglu et al.(2025) [26]	1. Prefabricated: PF (glass-fiber), PZ (zirconia), PT (titanium) 2. CAD/CAM: CF (glass-fiber), CZ (zirconia), CT (titanium	10 per group (total 60)– human maxillary central incisors	Universal testing machine, 45° angle, 0.5 mm/min	Thermal cycling (5000x, 5-55°C)	Prefab: Zr: 604 ± 134 N Ti: 537 ± 80 N GF: 444 ± 90 N CAD/CAM: Zr: 809 ± 126 Ti: 569 ± 123 N GF: 341 ± 66 N	Root fractures: 20-60% across groups. No significant difference between materials.	Zirconia posts (especially CAD/CAM) showed highest fracture resistance. CAD/CAM glass-fiber showed lowest.
17	Rodrigo Stadler Alessi et al. (2025) [27]	1.MW (moderate weakening): AFP (anatomical), MFP (CAD/CAM milled), UFP (universal 2-piece) 2. SW (severe weakening): same + control (NW-PFP)	10 per group (total 70) – human mandibular premolars	Compression, 150° angle, 0.5 mm/min (with crowns)	None	MW-AFP: 1043 ± 109.4 N MW-MFP: 1301.5 ± 113.7 N MW-UFP: 1199.2 ± 154.7 N SW-AFP: 996.7 ± 128.9 N SW-MFP: 1194.2 ± 109.3 N SW-UFP: 1128.6 ± 125.5 N Control (NW-PFP): 1077 ± 121.5 N	Predominantly repairable (type I: crown/post fracture). No catastrophic (type IV) fractures observed.	CAD/CAM milled and universal 2-piece posts showed superior fracture resistance to anatomical posts, with repairable failure modes.

CPC: cast post and core (metal); MFP: milled fiber post; GFP: (customized glass fiber + composite core); CAD/CAM: Computer-Aided Design/Computer-Aided Manufacturing; PICO: Population, Intervention, Comparison, Outcome; PRISMA: Preferred Reporting Items for Systematic Reviews and Meta-Analyses; PROSPERO: International Prospective Register of Systematic Reviews; MFP: Milled Fiber Post; PFP: Prefabricated Fiber Post; FRC: Fiber-Reinforced Composite; GFP: Glass Fiber Post; PICN: Polymer-Infiltrated Ceramic Network; PEEK: Polyetheretherketone; PEKK: Polyetherketoneketone; CPC: Cast Post-and-Core; HDP: High-Density Polymer; MPa: Megapascal; N: Newton; 3Y-TZ: 3 mol% Yttria-Stabilized Tetragonal Zirconia Polycrystal.

### Fracture strength outcomes

Fracture strength values varied considerably among the included studies owing to differences in materials, experimental designs, and testing protocols. Several investigations reported fracture strength values for CAD/CAM-fabricated glass fiber posts that were comparable to those of cast metal post-and-core systems. Pang et al. reported mean fracture strength values of 927.6 ± 275.6 N for CAD/CAM glass fiber posts and 967.9 ± 157.5 N for cast metal post-and-core restorations [5]. Similarly, Seckler et al. found comparable fracture strength values between CAD/CAM glass fiber posts and cast metal systems [23].

When compared with prefabricated fiber posts, CAD/CAM-fabricated glass fiber posts generally demonstrated comparable or higher fracture strength values. Gontijo et al. reported higher fracture strength values for milled glass fiber and PEEK post-and-core systems than for prefabricated fiber posts [21]. Alessi et al. also observed superior fracture strength in CAD/CAM milled posts compared with anatomical and prefabricated posts [27].

Comparisons among CAD/CAM materials revealed variable results. Rakotoaridina et al. reported higher fracture strength for milled glass fiber posts than for PEEK and polymer-infiltrated ceramic network materials [6]. In contrast, Hamitoglu et al. found zirconia-based CAD/CAM posts to exhibit the highest fracture strength values among the tested materials [26]. Several studies reported fracture strength values exceeding physiological anterior occlusal forces [10, 18, 21, 22, 27].

### Failure mode analysis

Failure mode was assessed in most of the included studies [5–7, 10, 13, 15, 18–21, 23–27]. CAD/CAM milled glass fiber post-and-core systems were predominantly associated with repairable failure patterns, including core fracture, post fracture, debonding, and cervical fractures located above the cemento-enamel junction [20, 21, 23, 27]. Pang et al. observed a greater number of repairable failures in the CAD/CAM glass fiber group compared with cast metal and prefabricated fiber post groups [5].

In contrast, cast metal and zirconia restorations more often led to catastrophic root fractures. Jrab et al. noted that all such failures occurred in the cast metal group, whereas CAD/CAM materials showed favorable failure patterns [20]. Likewise, Seckler et al. and Ibrahim et al. reported more repairable failures with CAD/CAM fiber‑reinforced posts than with rigid systems [23, 25].

### Effect of aging procedures

Eight studies incorporated aging protocols, including thermocycling, cyclic mechanical loading, or combined thermomechanical aging [5, 10, 18, 20–23, 26]. Aging procedures generally resulted in lower fracture strength values across all restorative systems. Nevertheless, CAD/CAM milled glass fiber posts maintained fracture strength values within clinically acceptable ranges after aging [18, 21, 23, 26]. Studies evaluating failure patterns following aging also reported that CAD/CAM fiber-reinforced restorations continued to exhibit predominantly repairable failure modes [15, 21, 23].

### Risk of bias assessment

The methodological quality of the included studies was evaluated using a modified CONSORT checklist adapted for in vitro studies [17]. Six studies were classified as having a low risk of bias [18–20, 23, 26, 27], while 11 studies were classified as having a moderate risk of bias [5–7, 10, 13–15, 21, 22, 24, 25]. No study was categorized as having a high risk of bias.

The most frequently fulfilled criteria were clear study objectives, standardized experimental procedures, clearly defined outcome measures, and appropriate statistical analyses. The most common methodological limitations were the absence of blinding procedures, lack of sample size calculation, and inconsistent implementation of aging protocols (Figure 2).

## Discussion

The present systematic review evaluated the fracture strength and mechanical behavior of CAD/CAM milled glass fiber post-and-core. The findings indicate that the biomechanical performance of these restorations is influenced by multiple interacting factors, including post-adaptation, elastic modulus compatibility, fiber orientation, manufacturing procedures, ferrule presence, and the integrity of the adhesive interface. Although methodological heterogeneity precluded quantitative synthesis, qualitative analysis allowed the identification of consistent biomechanical trends across the included studies.

One of the most recurrent findings among the included studies was the superior adaptation of CAD/CAM-fabricated posts to root canal anatomy compared with prefabricated systems. Customized CAD/CAM posts were associated with thinner and more homogeneous cement layers, which improved frictional retention and reduced interfacial discrepancies [5, 7, 21, 27]. Pang et al. described the resin cement as an essential component of the post-cement-dentin complex, contributing not only to retention but also to stress transfer within the restored tooth [5]. Similarly, Alessi et al. reported that milled fiber posts exhibited improved sliding frictional retention and superior canal adaptation, particularly in flared canals where prefabricated posts often require thick cement layers or relining procedures. Excessive cement thickness has been associated with void formation, polymerization contraction, and reduced bond stability [27], highlighting the importance of achieving a well-adapted post with a thin and homogeneous cement layer [5, 10, 21, 23, 27].

The elastic modulus of CAD/CAM fiber-reinforced materials also appears to play a major role in stress distribution. Several studies emphasized that CAD/CAM glass fiber materials exhibit elastic moduli closer to dentin, generally ranging between 25 and 35 GPa [5, 6, 19]. This biomechanical compatibility may reduce stress concentration within root dentin compared with rigid metallic or zirconia posts. Rakotoaridina et al. reported that materials with elastic properties closer to dentin demonstrated more favorable mechanical behavior, supporting previous observations by Dietschi et al. that dentin-like elasticity improves stress distribution along the root [6, 28]. In contrast, highly rigid materials such as zirconia and cast metal posts demonstrated higher fracture strength values in some studies but were more frequently associated with catastrophic and non-repairable root fractures [20, 26]. These findings emphasize that fracture strength should not be interpreted in isolation, as clinically favorable restorations should combine adequate load-bearing capacity with repairable failure patterns.

Fiber orientation and architecture were repeatedly identified as critical determinants of mechanical performance. Prachasujit et al. demonstrated that unidirectional fiber orientation parallel to the long axis of the post significantly improved flexural strength compared with multidirectional fiber arrangements [14]. Similarly, Gama et al. emphasized that proper alignment of fibers during CAD/CAM milling is essential to optimize stress dissipation and preserve mechanical integrity [13]. Although multidirectional fiber configurations may improve isotropic reinforcement, several authors reported that they may also reduce strength under unidirectional loading conditions [14, 15]. Ruschel et al. further demonstrated that the milling orientation directly influences fracture patterns, with vertically milled specimens exhibiting transverse fractures and diagonally milled specimens showing delamination and fiber separation [24]. These findings highlight the orthotropic behavior of CAD/CAM fiber-reinforced materials and confirm that mechanical behavior is highly dependent on fiber distribution and milling direction.

The integrity of the fiber-matrix interface also appears fundamental for the mechanical performance of CAD/CAM fiber posts. Eid et al. [7] and Fathey et al. [18] observed under scanning electron microscopy that failure of fiber-reinforced CAD/CAM materials typically begins within the resin matrix and propagates along the fiber–matrix interface, eventually resulting in sequential fiber rupture. This mechanism, commonly described as ‘brush-like cracking’, reflects progressive rather than catastrophic failure behavior [7, 18]. According to Ghanem et al. [19], the mechanical behavior of CAD/CAM fiber-reinforced posts depends not only on fiber orientation but also on the quality of bonding between the fibers and the resin matrix. Effective interfacial adhesion allows efficient stress transfer from the polymer matrix to reinforcing fibers, whereas interfacial defects may facilitate crack propagation and structural weakening [19].

The CAD/CAM manufacturing process itself may represent both an advantage and a limitation. The fabrication of monolithic one-piece post-and-core restorations eliminates the interface between the post and the composite core, thereby reducing potential sites of debonding and structural failure [7, 19]. However, several studies suggested that the milling procedure may compromise the integrity of the fiber network. Grandini et al., as discussed by Eid et al., reported irregular fiber surfaces and structural defects after milling procedures [7, 29]. Similarly, Hamitoglu et al. concluded that fiber cutting during milling may weaken the interface between fibers and the resin matrix, negatively affecting mechanical properties [26]. Ruschel et al. also observed that milling orientation affected crack propagation and delamination behavior [24]. Consequently, although CAD/CAM fabrication improves anatomical adaptation and eliminates the post-core interface, the mechanical performance of these restorations remains influenced by the quality of the manufacturing process and milling parameters.

Another important finding of the present review is the influence of ferrule effect and remaining tooth structure on fracture strength. Rakotoaridina et al. [6] emphasized that ferrule presence may be more important than the post material itself for long-term prognosis. A circumferential ferrule improves stress distribution and reinforces the cervical region of the tooth, reducing the risk of catastrophic root fracture [6]. Ibrahim et al. similarly reported that post length and ferrule design significantly affected fracture strength values. Although longer posts improved retention and stress distribution, excessive enlargement of the canal may weaken the remaining radicular dentin [25]. These findings suggest that the biomechanical performance of restored teeth depends not only on the post system but also on the preservation of adequate residual tooth structure, highlighting the importance of conservative post-space preparation.

Failure mode analysis revealed clinically relevant differences between CAD/CAM fiber posts and more rigid systems. Several studies demonstrated that CAD/CAM fiber-reinforced restorations were more frequently associated with favorable and repairable failures, such as core fracture, debonding, or cervical fracture above the cemento-enamel junction, whereas metallic and zirconia posts more often induced catastrophic vertical root fractures [20, 23, 25]. From a clinical perspective, this observation may be more relevant than fracture strength alone because repairable failures preserve the possibility of retreatment, whereas catastrophic root fractures frequently result in tooth loss.

Despite the promising biomechanical behavior of CAD/CAM glass fiber post-and-core, considerable heterogeneity was identified among the included studies. Differences in thermocycling protocols, mechanical aging procedures, ferrule dimensions, loading angles, post geometry, and testing methodologies complicated direct comparison between studies. Moreover, because all included studies were conducted in vitro, the overall level of evidence remains limited. Although several studies reported fracture strength values exceeding normal anterior occlusal forces [7, 18], these findings should be interpreted with caution, as static laboratory fracture tests cannot fully replicate the complex intraoral environment, where restorations are exposed to cyclic fatigue, multidirectional loading, thermal fluctuations, moisture, and biological factors. Consequently, the clinical performance of CAD/CAM glass fiber post-and-core systems cannot be inferred solely from in vitro fracture strength data, highlighting the need for standardized laboratory protocols and well-designed long-term clinical studies. In addition, this review was limited to studies published in English, which may have introduced language bias. The methodological heterogeneity and limited number of comparable studies also precluded quantitative synthesis, preventing the estimation of pooled effect sizes.

Overall, the findings of this review suggest that CAD/CAM-fabricated glass fiber post-and-core restorations represent a promising alternative to conventional post systems because of their improved adaptation, dentin-like elastic modulus, and more favorable failure behavior. Nevertheless, their mechanical performance cannot be attributed to a single material property but rather to a complex interaction between fiber architecture, adhesive integrity, manufacturing procedures, and preservation of tooth structure. Future research should prioritize standardized laboratory protocols and well-designed prospective clinical studies to enable more robust comparisons and strengthen the clinical evidence supporting CAD/CAM glass fiber post-and-core systems.

## Conclusion

CAD/CAM-fabricated glass fiber post-and-core systems demonstrated fracture strength comparable to conventional post systems in most included studies. Although a consistent superiority in fracture strength was not observed, these restorations generally exhibited more favorable and repairable failure modes than metal and zirconia post systems.

However, the available evidence is derived exclusively from in vitro studies with considerable methodological heterogeneity, limiting direct translation of these findings to clinical practice. Therefore, further standardized laboratory investigations and long-term clinical studies are required to confirm the clinical performance and longevity of CAD/CAM glass fiber post-and-core systems**.**

## Supplementary Material



## Data Availability

The data supporting the findings of this study are available within the article.
