# Supplementary material for: Fracture strength of CAD/CAM glass fiber post-and-cores: a systematic review
Source: Biomater Investig Dent. 2026 Aug 19;13:46724. doi: 10.2340/biid.v13.46724 (PMC13495068; doi:10.2340/biid.v13.46724)
Supplement: Supplementary file 1 [file BIiD-13-46724-s1.pdf]

Supplementary material has been published as submitted. It has not been copyedited or typeset by Biomaterial Investigations in Dentistry.

Appendix

Table A1

PubMed	((("CAD/CAM" OR "Computer-Aided Design"[Mesh]) AND ("glass fiber post" OR "glass fiber post and core" OR "post and core" OR "Post and Core Technique"[Mesh]) AND ("fracture resistance" OR "fracture strength" OR "Flexural Strength"[Mesh]))) OR ((("fracture resistance" OR "fracture strength" OR "Flexural Strength"[Mesh]) AND ("post and core" OR "Post and Core Technique"[Mesh])))
Scopus	((TITLE-ABS-KEY("CAD/CAM") OR TITLE-ABS-KEY("Computer-Aided Design")) AND (TITLE-ABS-KEY("post and core") OR TITLE-ABS-KEY("glass fiber post") OR TITLE-ABS-KEY("glass fiber post and core")) AND (TITLE-ABS-KEY("fracture resistance") OR TITLE-ABS-KEY("fracture strength"))) OR (TITLE-ABS-KEY("post and core") AND (TITLE-ABS-KEY("fracture strength") OR TITLE-ABS-KEY("fracture resistance")))
Web of Science	((TS=("CAD/CAM") OR TS=("Computer-Aided Design")) AND (TS=("post and core") OR TS=("glass fiber post") OR TS=("glass fiber post and core")) AND (TS=("fracture resistance") OR TS=("fracture strength"))) OR (TS=("post and core") AND (TS=("fracture strength") OR TS=("fracture resistance")))
Cochrane library	(("CAD/CAM" OR [mh "Computer-Aided Design"]) AND ("glass fiber post" OR "glass fiber post and core" OR "post and core" OR [mh "Post and Core Technique"]) AND ("fracture resistance" OR "fracture strength" OR [mh "Flexural Strength"]) OR ("fracture resistance" OR "fracture strength" OR [mh "Flexural Strength"]) AND ("post and core" OR [mh "Post and Core Technique"]))
